# A Survey on Energy Conserving Mechanisms for the Internet of Things: Wireless Networking Aspects

**DOI:** 10.3390/s151024818

**Published:** 2015-09-25

**Authors:** Zeeshan Abbas, Wonyong Yoon

**Affiliations:** Department of Electronics Engineering, Dong-A University, Busan 604-714, Korea; E-Mail: mars_zeeshan@yahoo.com

**Keywords:** Internet of Things, wireless access, energy conservation

## Abstract

The Internet of Things (IoT) is an emerging key technology for future industries and everyday lives of people, where a myriad of battery operated sensors, actuators, and smart objects are connected to the Internet to provide services such as mobile healthcare, intelligent transport system, environmental monitoring, *etc.* Since energy efficiency is of utmost importance to these battery constrained IoT devices, IoT-related standards and research works have focused on the device energy conserving issues. This paper presents a comprehensive survey on energy conserving issues and solutions in using diverse wireless radio access technologies for IoT connectivity, e.g., the 3rd Generation Partnership Project (3GPP) machine type communications, IEEE 802.11ah, Bluetooth Low Energy (BLE), and Z-Wave. We look into the literature in broad areas of standardization, academic research, and industry development, and structurally summarize the energy conserving solutions based on several technical criteria. We also propose future research directions regarding energy conserving issues in wireless networking-based IoT.

## 1. Introduction

The Internet of Things (IoT) has become an emerging key technology for future, in which a myriad of sensors, actuators, and smart objects in our daily life are connected to the Internet. These sensors and actuators (e.g., surveillance cameras, home appliances, and environment monitoring sensors) are typically equipped with different kinds of microcontrollers, transceivers, and protocols for communication of sensing and control data [1,2,3]. These real life objects, either sensors or actuators, are connected with each other to transfer their sensed data to centralized servers, where information is collectively stored and made available for particular users with proper access rights. The transfer of data from one sensor/actuator node to another sensor/actuator node or an IoT server is performed through a new communication paradigm called Machine Type Communications (MTC) or Machine-to-Machine (M2M). The communication technology for the first-hop of a path between an IoT device and an IoT server is mostly expected to be wireless radio access for the ease of installation and deployment. IoT devices use wireless radio access technologies such as Wireless Wide Area Networks (WWAN) and Wireless Local Area Networks (WLAN) to communicate with servers [4]. In some cases, constrained IoT devices may first communicate with intermediate entities called IoT gateways or M2M gateways through Wireless Personal Area Networks (WPAN) or WLAN. The gateways in turn forward data from these devices toward IoT severs, and act as a translator between IoT devices and servers [5,6,7,8]. Connectivity between IoT devices and other IoT gateways or servers can be provided by using different kinds of wireless technologies such as 3GPP Long Term Evolution (LTE) and LTE-Advanced, WiFi, ZigBee and Bluetooth or other standard wireless technologies [9,10].

The network characteristics of IoT using these wireless technologies are quite different from those for traditional wired or wireless networks because the number of devices participating in communication is very large. In addition, traffic per IoT device is typically not so much because each IoT device senses and transfers a small amount of data to a corresponding IoT server, although data generated from a huge number of objects may collectively have some impacts on the network performance. Furthermore, IoT networks should operate stably and sustainably for a longer period without any need for human intervention [11]. Another aspect is that gateways may incorporate multiple radio interfaces for versatile purposes such as throughput, latency, and energy efficiency [12].

Devices in such IoT networks will typically operate based on battery power sources, and hence, energy efficiency is naturally of utmost importance in device management. Looking into a particular Wireless Sensor Network (WSN) domain, energy efficiency for battery operated sensor nodes and lifetime prolongment have been research issues for so long [13,14], where Medium Access Control (MAC) layer protocols focus on adjusting the duty cycle for sensor nodes, and routing layer protocols are designed for data aggregation and many-to-one transmission. Similarly, since IoT devices operating in the IoT network paradigm are also battery operated, battery consumption should be kept in mind during IoT network deployment [15]. However, IoT network characteristics and deployment scenarios are more complex than traditional WSNs in various aspects, e.g., the volume of IoT devices, bidirectional traffic between IoT devices and servers, heterogeneous data for sensing and actuation, usage of heterogeneous wireless radio access technologies, existence of IoT gateways, *etc.* For this reason, some conventional WSN power saving strategies such as homogeneous data aggregation and clustering are not directly applicable to IoT cases. Extensive research is being conducted for energy conservation for battery-constrained IoT devices from various aspects such as standardization, academic research, and industry development.

In this paper, we present a comprehensive survey on recent efforts to resolve the energy conservation issues for resource-constrained IoT devices, and discuss issues and solutions provided in different kinds of literature. This survey examines the literature with a specific focus on wireless networking aspects for IoT energy conservation. The remainder of the paper is organized as follows. In Section 2, we provide basic information about IoT network architectures, IoT device structures, and various applications. In Section 3, we discuss some possible issues that can cause battery drainage of IoT devices and hence affect the lifetime of IoT devices and networks. In Section 4, we review energy conserving solutions provided in different kinds of literature. In Section 5, we propose important research directions regarding energy conserving issues for wireless networking-based IoT. In Section 6, we give concluding remarks.

## 2. Internet of Things: Network Architecture, Device Structure, and Applications

**Figure 1:**
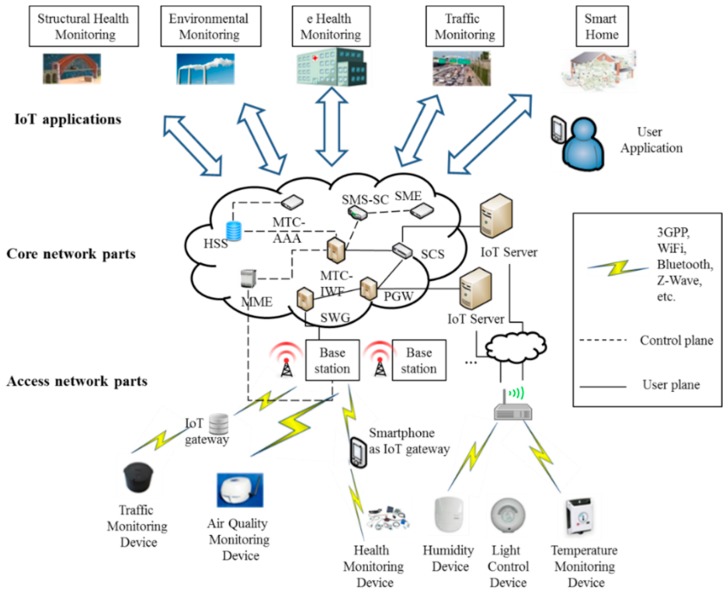
A generic Internet of Things (IoT) network architecture.

Figure 1 provides a generic view of an IoT network architecture using different wireless technologies, in which diverse IoT components are being connected to the 3GPP network components for proper operation and data transfer. Figure 1 shows the formation of IoT networks, also called M2M area networks, and also connectivity to IoT gateways and servers. The architecture presented in Figure 1 also represents a kind of a capillary network architecture, in which all devices are transferring their collected data to the IoT server through an intermediate entity that is an IoT gateway. Devices in an MTC domain typically transmit or receive a fixed amount of data in a fixed time interval. Inter-MTC device communication can be performed through wireless mobile networks or in an *ad hoc* network fashion [4,16,17,18,19,20,21,22,23,24,25,26]. In Figure 1, we can also notice particular application scenarios, where IoT devices and networks are being used for applications like structural health monitoring, environmental monitoring, human health monitoring [2,27], traffic monitoring [27,28], smart homes appliances [27], and so on.

**Figure 2:**
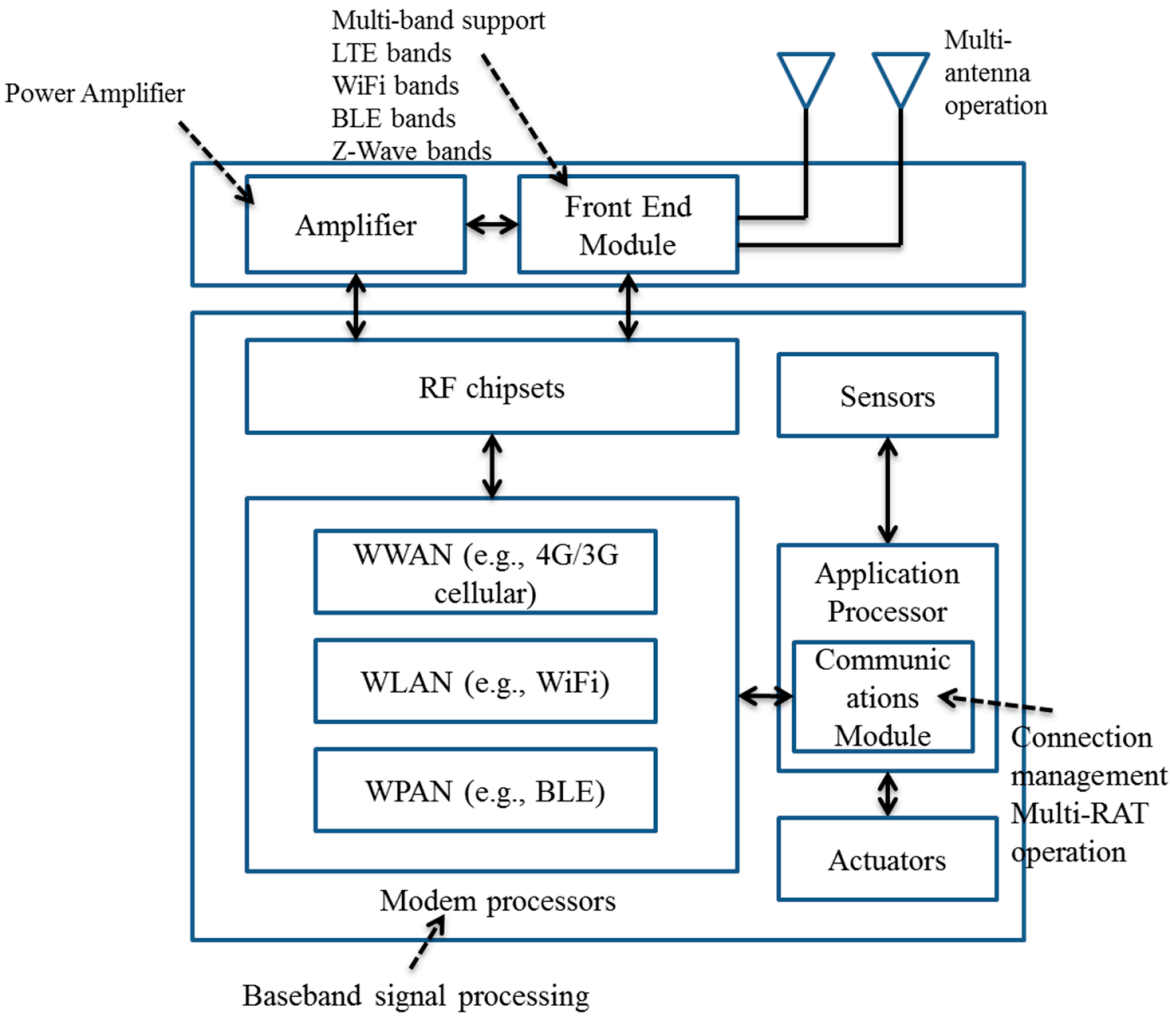
A generic IoT device/gateway structure in which different types of wireless technologies may be included.

In this IoT network architecture, we look into the further details of the IoT device or gateway structure as shown in Figure 2, where the necessary in-device components for transmission and reception of sensing and actuation data are illustrated. Considering an increasing need to support multiple heterogeneous Radio Access Technologies (RATs) for enhanced coverage and flexibility, Figure 2 provides a generic structure of multi-radio IoT devices or gateways with diverse communication components and their interaction with each other. Basically, an IoT device consists of different energy consuming communication modules (baseband chipsets, Radio Frequency (RF) chipsets, RF front end modules, antenna) that are collectively responsible for data communication over diverse radio access technologies [29]. To deeply understand the energy consumption behavior of IoT devices and gateways, we will need to consider the chain of in-device communication modules. In Figure 2, an IoT device or gateway includes sensor modules used for different sensing purposes such as temperature, light, *etc.* and actuator modules.

## 3. Energy Conserving Issues in Wireless Networking-Based IoT

**Figure 3:**
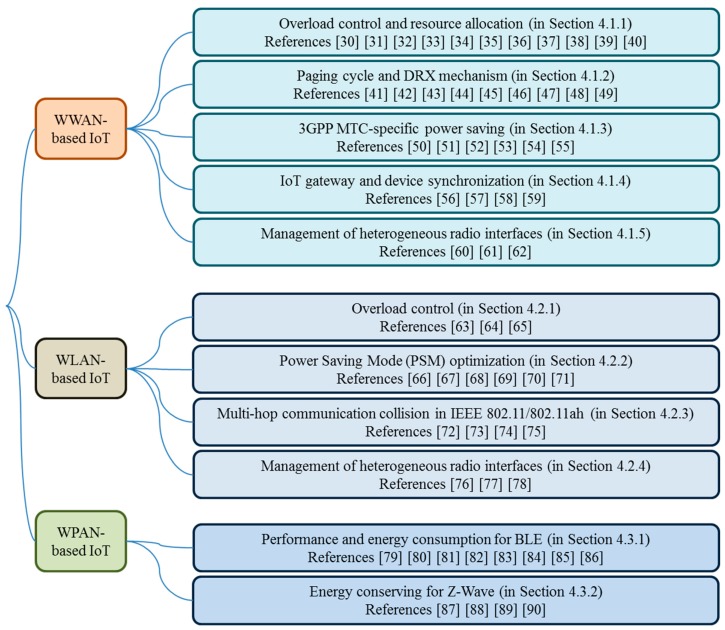
A taxonomy of IoT energy conserving issues for different types of radio access technologies.

Energy conserving issues or alternatively power saving issues in IoT devices during IoT/M2M network realization are closely related to particular wireless radio technologies used, e.g., 3GPP LTE/LTE-Advanced, WiFi, Bluetooth, or Z-Wave. Depending on the types of wireless radio access technologies, energy conserving issues arise in various manners, for example, how to control network overload or congestion, how to adjust duty cycles, how to allocate uplink or downlink radio resources in an energy efficient manner, *etc*. To guide what follows in the main body of this paper, we illustrate a taxonomy of energy conserving issues that we will review as shown in Figure 3. In this section, we will give a brief introduction to those issues for different types of wireless radio access technologies. The solutions to the issues and their categorization and comparison will be presented in more details in Section 4.

### 3.1. Energy Conserving Issues for WWAN-Based IoT

An important issue in using 3GPP LTE for IoT is attributed to a massive number of IoT devices. An overload or congestion problem in the radio access network as well as the core network arises when both user devices and IoT/M2M devices in LTE or LTE advanced networks try to access the network simultaneously for data transfer. The problem can be severer as the number of devices, either Human-to-Human (H2H) or Machine-to-Machine (M2M), will increase in upcoming years. The overload problem can indirectly affect energy consumption in IoT devices because the overload condition, and hence high network utilization may cause delay and loss for IoT data packets and retransmitting them may incur more battery power consumption [30,31,32,33,34,35,56,91]. In addition to congestion-induced energy consumption issues, another important factor is an energy-efficient allocation of radio resources (in terms of time, frequency, and transmit power) to IoT devices [36,37].

**Figure 4:**
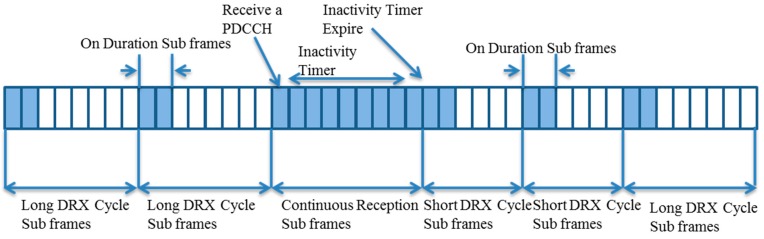
A basic 3GPP DRX mechanism.

To manage the power consumption of user nodes, mostly smartphones, the 3GPP has defined a conventional mechanism called Discontinuous Reception/Transmission (DRX/DTX) cycles or paging cycles. Figure 4 illustrates a basic DRX mechanism. The DRX mechanism is further classified into Connected DRX for devices in a connected state and Idle DRX for devices in an idle state. On every On-Duration period, a device wakes up and checks Physical Downlink Control Channel (PDCCH) scheduling information in subsequent subframes (one subframe is 1 ms long). If the device is not scheduled, it goes back to a sleep mode for low power operation. Otherwise, it will stay in an active mode to receive or send its data and start the Inactivity timer. If the Inactivity timer expires with no data transmission or reception, the device enters short DRX cycles. An issue is that conventional duty cycling may not be adequate for IoT and may possibly cause battery drainage problems for IoT/M2M devices [41,42,43]. Conventionally, the 3GPP has limited a maximum paging cycle to be 2.56 s. However, this limit is not adequate for IoT/M2M devices, as they require very infrequent data transmission compared with human type communications. Some DRX parameters may have major or minor impacts on the power consumption of IoT devices [44,45,46]. Paging messages sent from eNBs to UEs can also bring up energy consumption in IoT devices.

In the 3GPP standards, significant efforts have recently been made to develop standard mechanisms and protocols for MTC support in cellular networks [47,50,51,52]. They discussed various MTC-specific issues (e.g., transmit power, bandwidth, duplex operation, peak rate, and downlink transmission modes) that can have impacts on device power consumption and specified solutions for them. Other issues may include the difficulty of synchronization or connection establishment between IoT/M2M devices and IoT/M2M gateways for proper data transfer, which could adversely affect power consumption [53,54,57,58,59], and the usage of multiple heterogeneous radio interfaces.

### 3.2. Energy Conserving Issues for WLAN-Based IoT

**Figure 5:**
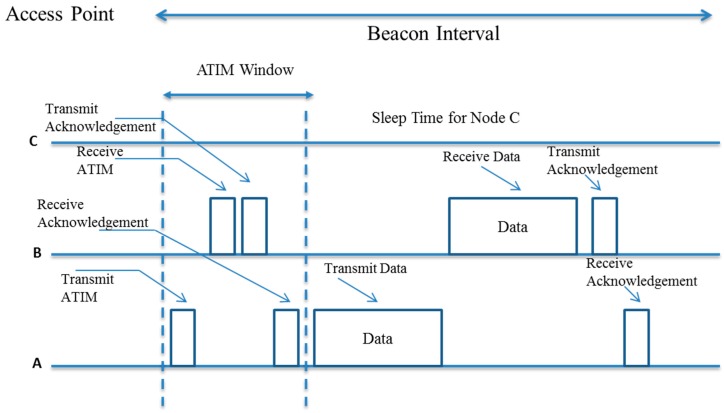
An illustration of IEEE 802.11 PSM.

Basic energy conservation for WiFi devices is achieved through IEEE 802.11 Power Save Mode (PSM) as illustrated in Figure 5. In *Ad hoc* Traffic Indication Map (ATIM) window after the start of each beacon interval, a node remains awake for the exchange of ATIM requests and responses. A node sends an ATIM request message if it has a packet to send to an intended receiver. The receiver sends acknowledgement and will stay awake during the rest of the beacon interval for packet reception. Otherwise, it can go into a sleep mode for power saving. The existing IEEE 802.11 PSM may not be adequate for battery-operated constrained IoT devices and multi-hop IoT network settings [66,67].

In the case that IoT devices use WiFi to reach the 3GPP core network and eventually IoT servers, device power consumption due to congestion in WiFi networks should also be kept in mind [63,64,65]. When IoT devices use WLAN type technologies such as the IEEE 802.11 family standards, they may construct multi-hop topologies to enlarge their reachability. In such cases, there may arise the issue of increased power consumption due to severe collisions in multi-hop communications [66,67]. The standardization efforts, *i.e.*, IEEE 802.11ah, are being made to make a more suitable choice for IoT. Collision avoidance schemes and their implementation regarding the multi-hop communication collision issue are necessary for IEEE 802.11ah [72,73].

### 3.3. Energy Conserving Issues for WPAN-Based IoT

**Figure 6:**
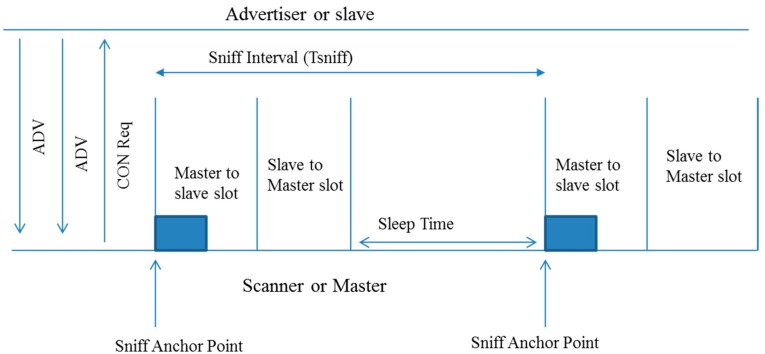
An illustration of Bluetooth Low Energy (BLE) Sniff mode.

Bluetooth Low Energy (BLE) is one of the most promising low power consumption wireless technologies in the wireless personal area networking domain for IoT applications. Figure 6 illustrates the duty cycle of a slave device by using the Sniff interval method during which it listens to a master device for a particular amount of time. At every Sniff anchor point (typically every 100 ms), the device wakes up to check if it has data to send or receive, and goes back to a sleep mode for the rest of sniff interval. The issue of determining the sleeping time of BLE devices according to a battery level and throughput-to-workload ratio is examined in [83].

Protocol mechanisms for using BLE for IoT, and their relationships with energy consumption issues have been examined, such as the relationship between header compression and energy consumption, the impact of using IoT gateways as proxies on energy consumption, relationship between duty cycling and energy consumption, routing mechanisms for BLE energy consumption, and the influence of IoT data delivery protocols on energy consumption, in the literature [79,80,81,82,92]. Particularly, the usage of multicast signaling messages for IPv6 neighbor discovery over BLE assumes that the message receivers are powered on to be able to receive the messages, which can require energy consumption.

Z-Wave is an emerging wireless protocol for smart homes, where the Z-Wave based devices can be remotely controlled. Basic considerations for Z-Wave for home automation systems include how to manage devices in a home environment in a power efficient way [87,88]. Fault management in Z-Wave is also related to energy consumption [89]. More specifically, if some devices fail in their operation, other devices that continuously try their data transmission through those failed devices would end up with huge energy consumption.

## 4. Energy Conserving Solutions in Wireless Networking-Based IoT

Having briefly looked at important energy conserving issues for IoT in the previous section, we now discuss the solutions proposed in the literature for different wireless access technologies. The following three Sections (Section 4.1, Section 4.2 and Section 4.3) will deal with energy conserving solutions for WWAN-based IoT, WLAN-based IoT, and WPAN-based IoT, respectively. In each subsection, we will review solutions to issues with criticism, and also summarize the survey in a table form for a comparison purpose, in terms of categories, approaches, and various technical criteria such as schemes (types of algorithms and mechanisms used), metrics (performance metrics of interest), control (who controls the mechanisms), and evaluation (performance evaluation methods).

### 4.1. Energy Conserving Solutions for WWAN-Based IoT

First of all, we will review energy-conserving solutions for IoT using a representative WWAN, *i.e.*, 3GPP LTE. These solutions include: overload control for the access and core networks and energy-efficient radio resource (time, frequency, and transmit power) allocation, paging cycle or DRX cycle optimization, MTC-specific energy conserving, resolving gateway-device synchronization, and handling multiple heterogeneous radio interfaces.

#### 4.1.1. Solutions for Overload Control and Radio Resource Allocation

In this subsection, we will discuss the solutions for the overload or congestion issue. Cheng *et al.* [30] discussed push and pull based methods to overcome the overload problem in the radio access network. They also proposed methods to alleviate the congestion problem in the core network, including the use of extended access barring, extended wait timer, and delay tolerant indicator. Laya *et al.* [31] presented random access techniques to avoid the congestion and overloading problem that massive IoT/MTC devices face in the 3GPP network environment. They developed more energy efficient mechanisms for the existing baseline techniques, e.g., access class barring, back-off adjustment schemes, dynamic allocation of Random Access Channel (RACH) resources, prioritized random access, *etc*. Zheng *et al*. [32] provided a solution to signaling message storms like RACH messages in the presence of both Human Type Communications (HTC) and MTC. The solution is to split HTC and MTC devices into two groups, and an evolved NodeB (eNB) makes prioritization decisions to grant access according to a type of a device. For example, if a collision occurs because of MTC devices, then a priority will be given to HTC devices. Abdalla *et al.* [37] proposed that available radio resources in terms of time and frequency, called Resource Blocks (RBs), should be divided into two groups, *i.e.*, a UE specific set dedicated to UEs and an M2M specific set dedicated to M2M devices. Separate Quality of Service Class Indicators (QCIs) are defined for both types of devices so that user quality of experience should not be affected in the presence of different types of devices.

The proper resource allocation of Modulation and Coding Scheme (MCS) and transmit power is a key factor to energy efficient communications. Wang *et al.* [33] proposed an energy efficient solution for optimal MCS determination and LTE uplink power control, and implemented it to transfer MTC data over an LTE network. Silva *et al.* [36] noted that devices in an LTE network send signaling messages for downlink or uplink channel condition assessment to their eNB, which has to make a scheduling decision in every 1 ms. To resolve the problem, they suggested that when an eNB notices any performance degradation by employing statistical and machine learning techniques, it can instruct devices to stop reporting downlink or uplink related channel information to save their power. Chen *et al.* [34] proposed the usage of LTE–Advanced relays and focused on uplink resource and transmit power allocation to save the energy of MTC devices. In the presence of LTE-Advanced relays that can enhance radio coverage and data rates, and increase the throughput of cell edge devices, they proposed optimal MCS and transmit power allocation for energy efficient communication through the relays.

In the discussed solutions, we notice that decisions for overload control and resource allocation are only determined by a single eNB. More optimal decisions can be possible if a group of eNBs share their information about camping-on MTC devices to make global information.

#### 4.1.2. Solutions for Paging Cycle Optimization

For efficient power saving in 3GPP networks, some works have looked into LTE paging cycle mechanisms (also known as DRX cycles). Tang *et al.* [41] proposed the concept of self-adaptive DRX, which is based on the adaptive selection of normal and extended DRX cycles. Using a self-adaptive DRX technique, an IoT device has to wake up twice to listen to a paging message. The length of these two mixed normal and extended DRX cycles is fixed, but it can be adjusted later according to the last values. Jha *et al.* [42] provided a solution that exploits the nature of infrequent IoT data transmission, possibly using the extended DRX cycles, which can result in increased power saving. Power saving can also be improved by decreasing the Radio Resource Control (RRC) inactivity timer for any paging cycle. A DRX optimization solution with QoS consideration is proposed in [43], in which firstly the values of short DRX, long DRX, the DRX inactivity timer, and the DRX start offset are calculated by an eNB and assigned to each User Equipment (UE). All UE wakeup periods are an integer multiple of others. Next, the DRX inactivity timer is optimized by reducing the timer value. As a third step, devices should go to a deep sleep state, when there is no data transmission or reception. By using the proposed solution, significant energy saving can be achieved while guaranteeing QoS.

To improve the paging mechanism, Jeong *et al.* [44] provided a solution to allocate larger DRX cycles for UE High Power Saving Reception Mode (HPSRM) as compared to UEs in normal modes. Luft *et al.* [45] proposed so-called a stateful paging guard device that is used to determine the state of terminal devices, either reduced energy consumption states or active states. To improve device standby time, Ramchandran *et al.* [46] proposed including information such as session interval and a device’s connection state indicators in signaling messages from the network. Based on the information contained in the signaling messages, a device will remain in an idle state, connected state, or low power state for a particular amount of time instructed by the network. Jha *et al.* [57] proposed turning the LTE radio processor off, instead of going to an idle state. This can result in significant power saving when there is no data transmission. A framework for application-assisted power saving is proposed in [58]. For instance, if a user application wants to access any network, it first checks the radio state of that device. If the device is in a sleep mode, then it buffers request messages (in a delay tolerant way) and will transmit messages on device’s next wakeup.

3GPP introduced the concept of low cost MTC (LC-MTC) terminals to reduce power consumption in LTE networks. In [47], different methods have been discussed for UE power consumption optimization, including extending DRX in an idle mode, extending DRX using UE assistance information, and power saving states for devices. The standard document [52] provides information about the factors which can affect power consumption of IoT devices, for example, the reduction of maximum bandwidth, a single receive RF chain, the reduction of transmitting power, half duplex operation, the reduction of the peak rate, and the reduction of supported downlink transmission modes. Tirronen *et al.* [48] presented a method to reduce the energy consumption of M2M devices in an LTE network. They considered the DRX mechanism for reducing the energy consumption of devices during active and non-active periods. By modeling different parameter settings for M2M devices, an energy consumption analysis is performed. The results of this analysis indicate that making the current DRX cycle period longer will reduce energy consumption in M2M devices. Paging messages sent from eNBs to UEs can also be a cause for energy consumption in H2H or M2M devices. To handle the amount of these IoT/M2M paging messages, Chao *et al.* [93] proposed a three layered IoT/M2M paging mechanism. At the first layer Paging Occasion (PO) for IoT/M2M devices is defined and calculated by devices. At the second layer devices are identified by their device identity, and certain devices can be paged by using their device identity’s to wake up in certain POs. At the third layer, a reason for paging is defined to show why the certain device has to wake up.

Paging cycle mechanisms will surely contribute to most of energy conservation in IoT devices. However, there is a fundamental tradeoff between energy saving and transfer delay. As IoT more spans its application areas to more mission critical and delay sensitive ones, e.g., emergency alarming and health care in hospitals, further research is needed to balance between energy conserving and responsiveness.

#### 4.1.3. Energy Conserving Solution for 3GPP MTC Devices

3GPP has specified diverse power saving schemes for MTC devices. In a 3GPP standard [46], UE Power Saving Mode (PSM) is introduced in which a UE may remain registered to the core network with no need to re-attach or re-establish connection with the core network. The UE will be active only during the exchange of signaling messages such as Tracking Area Update (TAU) and then the TAU procedure will take place between M2M gateways and the core network. However, we think that device triggering for a UE in a deep sleep mode needs further studies. In a 3GPP standard [51], it is suggested that an MTC device can transfer data only after it has performed a TAU procedure. At that time, the device has transitioned to an active state from a previous sleep state, and is ready to receive or transmit data for a particular allowed time. 3GPP also discussed an optimized TAU signaling mechanism for a case where a current configured TAU timer causes some congestion problems. The timer may be reconfigured by Mobility Management Entity (MME), and then, a detach procedure and a re-attach procedure are followed to allocate the new timer to the MTC devices.

Another energy consumption issue is related to a connection establishment procedure. Batchu *et al.* [53] suggested suppressing explicit network registration or attachment procedures for certain M2M devices to save their power. An M2M device has to select a radio access network (RAN) from available networks and then it will capture and transmit sensing data to a corresponding M2M server without any need for explicit registration. After transmission, it will go to a low power state for a particular amount of time. Later, when the device wakes up, it selects a RAN and carries out the same procedure. A power saving mechanism in which a device can transfer data during an initial connection establishment procedure is suggested in [54]. After the device can access a network, it will append collected sensing data to a bandwidth allocation message called a network entry message. If data are successfully transmitted, then the procedure is completed. Otherwise, it will retry the procedure for predetermined times or has to establish a connection to transmit data.

#### 4.1.4. Solutions to Gateway and Device Synchronization Problem

Singh *et al.* [56] considered a particular scenario where LTE and WiFi networks coexist in an M2M environment, and suggested device-gateway synchronization as an energy efficient solution. An IoT gateway-calculated Listen Interval (LI) is sent to IoT devices so that they can update their wake up intervals with respect to the gateway. As an industry development, Singh *et al.* [59] proposed a mechanism that supports M2M device and gateway synchronization, in which the gateway calculates listen intervals for particular devices and transmits that information in a beacon format to these devices. Receiving the beacon-like message, the devices update their listen interval and accordingly remain in a sleep mode as per the updated interval to save energy. However, we notice that if the device is in sleep mode, and gateway receives some requests from some application, then it has to wait for the device to wake up. In this regard, this solution becomes not applicable for delay sensitive applications.

#### 4.1.5. Energy Conserving Solutions for IoT Devices with Heterogeneous Radio Interfaces

The intelligent support of radio interfaces in operation is a key technical issue for multi-radio equipped devices in general. We envisage that future IoT devices will also incorporate multi-radio interfaces and power consumption for driving the multi-radio interfaces will be one of technical issues. With this in mind, we survey some previous works on energy conserving for multi-radio devices in general although not designed specifically for IoT. Andreev *et al.* [60] proposed a solution for energy saving by intelligently selecting an operating interface in such multi-radio enabled device. The criteria for energy efficient interface selection can be based on various values such as predefined Signal-to-Noise Ratio (SNR) thresholds, network load, or throughput. Parallel Wireless [61,62] proposed support for multiple radio access technologies, where relay nodes manage multiple radio interfaces in a self-organizing manner as controlled by a server in a cloud. The packets from relay nodes are transmitted based on their priority, and route selection for the packets is done by Self-Organizing Network (SON) module according to a receiver’s conditions such as interference and SNR on each particular radio. However, we think that there is a strong need for this technique to be deployed in a real world for experimental evaluation of how heterogeneous interfaces will switch dynamically during run time and energy efficiency as well. Shah *et al.* [94] proposed a handover mechanism for heterogeneous multi-radio devices that can also have an impact on device power consumption. They devised an algorithm for switching between radio interfaces to support QoS requirement.

#### 4.1.6. Summary

Table 1 summarizes the extant energy conserving solutions for WWAN-based IoT in terms of categories, approaches, and various technical criteria such as schemes, metrics, control, and evaluation.

### 4.2. Energy Conserving Solutions for WLAN-Based IoT

When IoT devices are connected to the Internet through a representative WLAN, *i.e.*, IEEE 802.11 WiFi network, power consumption for driving the WiFi interface should be controlled in an energy conservative manner. The solutions we will review include: overload control for the access network, IEEE 802.11 PSM optimization, resolving multi-hop communication collisions, and handling multiple heterogeneous radio interfaces.

**Table 1:** Energy conserving solutions for wireless wide area network (e.g., 3GPP)-based IoT.

Category	Solution	Approach	Scheme	Metric	Control	Evaluation
Overload control, resource allocation	[30,31,32]	Extended access baring (EAB), extended wait timer and delay tolerant indicator, prioritization mechanism	Back off mechanism	Energy efficiency, access delay, access probability	Distributed	Simulation
	[95]	Extending EAB to four paging cycles	Back off mechanism	Energy efficiency, admission rate	Distributed	Simulation
	[33]	Optimal MCS determination and transmit power control	MCS adaptation, transmit power control	Energy efficiency	Distributed	Simulation
	[35]	Clustering devices and efficient resource allocation	Resource allocation	Energy efficiency	Centralized/Distributed	Simulation
	[38]	Reinforcement learning algorithm based eNB selection	Back off counter and algorithm	Energy efficiency, access probability	Distributed	Simulation
	[39]	Small cell based traffic handling using HeNB	Small cell, HeNB for MTC	Energy efficiency	Centralized/Distributed	Simulation
	[40]	Avoiding near-simultaneous network entry attempts with larger back off values	Back off mechanism	Energy efficiency, access probability, access delay	Distributed	Simulation
	[36]	Statistical methods based reference signal stopping	Back off mechanism	Energy efficiency	Centralized/Distributed	Experiment
	[37]	UE and M2M devices differentiation based resource allocation	Resource allocation	Energy efficiency	Centralized	Experiment
Paging cycle, DRX mechanism	[42,43]	Extending paging cycle	Duty cycling	Energy efficiency, end-to-end delay	Centralized/Distributed	Analysis, simulation
	[48]	Longer DRX cycles for LTE devices	Duty cycling	Energy efficiency, reporting interval	Centralized/Distributed	Simulation
	[47]	Extended DRX mechanism	Duty cycling	Energy efficiency	Centralized/Distributed	Qualitative analysis
	[44]	Increased DRX cycle of devices operating in HPSRM	Duty cycling	Energy efficiency	Centralized/Distributed	Experiment
	[45]	State determiner introduced to determine state of devices to save power	Duty cycling	Energy efficiency	Centralized/Distributed	Experiment
	[46]	Network assisted standby timer for MTC devices	Duty cycling	Energy efficiency	Centralized/Distributed	Experiment
	[49]	Service-Instance Oriented Energy Management	Duty cycling	Energy efficiency	Centralized	Experiment
3GPP MTC-specific power saving	[50]	UE Power Saving Mode	Duty cycling	Energy efficiency	Distributed	Qualitative analysis
	[51]	Allowed time period for sleep after TAU/RAU	Duty cycling	Energy efficiency	Centralized/Distributed	Qualitative analysis
	[52]	Reduction in maximum bandwidth, transmit power and half duplex operation	Transmit power control	Energy efficiency, end-to-end delay	Centralized	Qualitative analysis
	[53]	RSSI based connection establishment	Transmit power control	Energy efficiency, end-to-end delay	Distributed	Experiment
	[54]	Device data transfer during initial communication	Transmit power control	Energy efficiency	Distributed	Experiment
	[55]	Energy efficient long range data transfer	Transmit power control	Energy efficiency	Distributed	Experiment
Gateway and device synchronization	[56,59]	Gateway calculated LI based device synchronization	Duty cycling	Energy efficiency, packet transfer time	Centralized	Simulation
Energy conserving management of heterogeneous radio interfaces	[60]	SNR, throughput and RSSI level based interface selection	Transmit power control	Energy efficiency, end-to-end delay	Centralized/Distributed	Simulation
	[61]	Controlled interface switching based on environmental parameters	Transmit power control, interference, congestion	Energy efficiency	Centralized/Distributed	Experiment
	[62]	Controlled interface switching based on environmental parameters	Transmit power control, interference, congestion	Energy efficiency	Centralized/Distributed	Experiment

#### 4.2.1. Solutions to Overload Condition

Overload or congestion is one of the major challenges in WLAN-based IoT as well as in WWAN-based IoT. In this regard, Liu *et al.* [63] proposed an offset listen interval algorithm to alleviate network contention and delay. The algorithm ensures that M2M traffic be spread evenly in WLAN beacon periods with calculated offsets and Access Points (APs) make wake-up schedules in such a way that a minimum number of devices wake up during a certain beacon period. However, we think that there needs some enhancement to assure different delay bounds for delay-sensitive IoT devices for emergency applications. Lin *et al.* [64] suggested a deep sleep mechanism that enhances IEEE 802.11 PSM by granting a higher channel access priority for low-energy devices dynamically. The mechanism resolves the overhearing or congestion problem as device wake-up time is randomly deferred. IoT devices have to wake up after a certain amount of time and then an IoT gateway will transmit all buffered packets to all the devices. An analysis of energy and delay under two contention resolution mechanisms, *i.e.*, frame slotted Aloha and tree splitting contention resolution is presented in [65]. Based on the optimal packet length, the mechanisms assure energy efficiency and minimum delay for M2M networks.

#### 4.2.2. Solutions to Optimize IEEE 802.11 Based PSM

The existing IEEE 802.11 PSM may not be adequate for battery-operated IoT devices and multi-hop IoT network settings. Authors in [66,67] extended the existing PSM to a multi-hop PSM in which traffic announcements are propagated along multi-hop wireless links and all intermediate devices should stay awake to forward received data with minimum latency. The scheme can improve end-to-end delay and enlarge device sleep time. Chen *et al.* [68] suggested mobility supported PSM that considers both user mobility and traffic conditions for making sleep/wakeup schedules. Based on channel condition estimation, APs buffer packets and mobile stations adjust their sleep interval to reduce wake-up overhead. However, we think that consideration for delay aspects in sleep interval adjustment is needed for delay-sensitive IoT. Wang *et al.* [84] examined the usage of low-power WiFi modules for providing IP connectivity for sensors deployed in various IoT scenarios. Particularly, battery lifetime depends heavily on scenarios, e.g., frequent wake-up to receive timely command messages, periodic data transmission, connection maintenance, *etc.*

#### 4.2.3. Solutions for IEEE 802.11/802.11ah Multi-Hop Communication Collision

While using IEEE 802.11ah in IoT networks, Ogawa *et al.* [72] and Adame *et al.* [73] introduced a collision avoidance mechanism called virtual grouping for Carrier Sense Multiple Access/Collision Avoidance (CSMA/CA). Based on a random Arbitration Inter-Frame Space Number (AIFSN) scheme, they support two types of stations (STAs), *i.e.*, contending STAs and non-contending STAs. After data transmission, an STA obtains a new AIFSN value. If the obtained value is lower than a predefined threshold, then the STA is placed in contending STAs. Otherwise, it is placed in the block of non-contending STAs. Power saving is achieved by moving non-contending STAs to a sleep mode. However, the overhead of dynamic grouping based on data availability should consume additional energy but it is not considered. Sun *et al.* [74] proposed a packet buffering strategy to store packets at an AP if devices are in a sleep mode. The AP sends a message to such sleeping devices during specific intervals based on their category, either Traffic Indication Map (TIM)-based or non-TIM-based. Packets are then transmitted only to TIM-based devices while all non-TIM devices are kept in a sleep mode to further save power. Power saving mechanisms can also be deployed on Android smart phones. Bernardo *et al.* [69] presented an end user power saving control in Android devices in which they extended Android framework for power saving control and moved that control to end users. The methodology provides end users with the application control of the IEEE 802.11 interface power management so that different energy saving schemes can be implemented. Raeesi *et al.* [75] proposed a collision avoidance mechanism called Restricted Access Window (RAW) mechanism. The mechanism allows an AP to allocate a medium access period, which is divided into one or more time slots. RAW information is carried in beacons transmitted by AP. Time slots are assigned to a group of STAs and they are allowed to access the medium accordingly, during the allocated slots. As a result, chances of collision get reduced and power saving for devices can be achieved.

#### 4.2.4. Energy Saving Solutions for IoT Devices with Heterogeneous Radio Interfaces

Although not intended specifically for IoT, there have been some previous works on energy conserving for multi-radio devices including WiFi. Bahl *et al.* [76] discussed power saving for multiple interfaces enabled devices and introduced the use of a Low Power Radio (LPR) in devices. When a device is not transmitting or receiving data, it shuts down a High Power Radio (HPR) and activates the LPR, and accordingly offloads data from the HPR to the LPR. Friedman *et al.* [77] introduced a management middleware for device power saving, in which devices not present in an overlay network can switch their radios off to save power and can wake up only when they are present in an overlay network. The major motivation for supporting heterogeneous interfaces can be found in Qin *et al.* [78]. They proposed a ZigBee assisted WiFi transmission system where ZigBee-based clustering is used to coordinate the communication turns of WiFi to reduce contention and collision. According to the system, the existence of WiFi networks is checked based on information provided by a ZigBee interface and packets are transmitted and received using a WiFi interface. However, it is not clear whether the proposed system outperforms other proposals for WiFi PSM. Considerations of wireless channel conditions such as SNR and interference should also be further investigated to enable proper usage of heterogeneous radio interfaces.

#### 4.2.5. Summary

Table 2 summarizes energy conserving technical issues for WLAN-based IoT, and solutions in terms of categories, approaches, and technical criteria such as schemes, metrics, control, and evaluation.

### 4.3. Energy Conserving Solutions for WPAN-Based IoT

WPANs are expected to play a crucial role as an energy efficient connectivity for battery-powered constrained IoT devices, with Bluetooth being one of the widely deployed WPANs. We will discuss the use of Bluetooth and Bluetooth Low Energy (BLE) implementation for energy efficiency in IoT scenarios. We will also discuss Z-Wave, a widely used technology in home environments.

**Table 2:** Energy conserving solutions for wireless local area network-based IoT.

Category	Solution	Approach	Scheme	Metric	Control	Evaluation
Overload control	[63,96]	Calculated offset listen interval spread among devices	Duty cycling	Energy efficiency	Centralized	Analysis, simulation
	[64]	Higher channel access priority to low energy level devices	Device power	Energy efficiency	Distributed	Algorithm, simulation
	[65]	Frame slotted aloha and tree splitting algorithm based duty cycle synchronization	Duty cycling	Energy efficiency, end-to-end delay	Centralized/Distributed	Analysis, simulation
Power Saving Mode (PSM) optimization	[66,67]	PSM and traffic announcement extension for IEEE 802.11	Duty cycling	Energy efficiency, end-to-end delay	Distributed	Simulation
	[68]	Mobility supported PSM	Duty cycling	Energy efficiency, end-to-end delay	Distributed	Simulation
	[70]	Schedule-aware PSM	Duty cycling	Energy efficiency	Centralized/Distributed	Analysis, simulation
	[69]	IEEE 802.11 android power saving framework extension	Transmit power control	Energy efficiency, end-to-end delay	Distributed	Experimental test bed
	[71]	New low power WiFi chip/modules	Transmit power control	Energy efficiency, interference, communication range	Distributed	Real time experiment
Solutions for IEEE 802.11/802.11ah multi-hop communication collision	[72]	Virtual grouping for contending and non-contending STAs	Contention alleviation	Energy efficiency	Distributed	Algorithm, simulation
	[73]	Performance analysis of IEEE 802.11ah	Transmit power control	Energy efficiency, transmission range, data rate, end-to-end delay	Distributed	Real time experiment
	[74]	Packets buffering strategy for TIM and non-TIM devices	Duty cycling	Energy efficiency	Centralized/Distributed	Simulation
	[75]	Restricted access window mechanism	Duty cycling	Energy efficiency, throughput, end-to-end delay	Distributed	Analysis, simulation
Energy saving management of heterogeneous radio interfaces	[69]	Low power radio based interface energy management	Duty cycling	Energy efficiency	Centralized/Distributed	Simulation
	[77]	Middleware based on overlay module for interface management	Duty cycling for devices in overlay network	Energy, end-to-end delay and capacity	Centralized	Simulation
	[78]	ZigBee assisted WiFi transmission	Congestion control	Energy efficiency	Centralized	Simulation

#### 4.3.1. Energy Conserving Solutions for Bluetooth Low Energy (BLE)

Bluetooth Low Energy is a widely used WPAN technology in various IoT scenarios. Siekkinen *et al.* [92] and Gomez *et al.* [82] presented a BLE implementation and performance evaluation in comparison to ZigBee/802.15.4. Energy is spent during a master-slave discovery process when a master device and slave devices are not in a connected state and the master device scans for available slaves to connect and at the same time slave devices advertise their availability to the master. Energy spent after connection establishment is also considered in the analysis where energy consumption related parameters such as data transmission, data reception, and inter-frame spaces are examined.

Using BLE with IPv6 support is considered essential in connecting a myriad of IoT devices to the Internet. Authors in [79,80,81] discussed energy-efficient neighbor discovery, header compression, and fragmentation to enable IPv6 on top of Bluetooth, and evaluated their impact on energy consumption. They showed the existence of a tradeoff between latency and energy consumption, which depends on device connection intervals. Particularly, a neighbor discovery mechanism for IPv6 over BLE is the major cause for energy consumption of BLE-based IoT devices. A huge number of multicast messages transmitted may result in severe energy consumption for BLE devices. To overcome this issue, the authors proposed an optimized neighbor discovery mechanism that achieves a reduced number of multicast message transmissions by using node registration and neighbor cache management. During a node registration process, a lifetime for the node is chosen in the neighbor cache. When the node’s lifetime expires, it is removed from the neighbor cache. Basically, node registration entries are kept in the neighbor cache to reduce the transmission of neighbor solicitation messages from other nodes.

A middleware solution to reduce the size of protocol stacks and network footprints affecting device energy consumption is proposed in [85]. For this purpose, a compressed header is formed into a frame at IoT devices, and it is restored at IoT gateways to obtain the original frame. Collotta *et al.* [83] proposed a fuzzy logic controller on a BLE master node that calculates new values of the sleeping time for each connected BLE device. The fuzzy logic controller calculates the sleeping time of each device according to the battery level and the ratio of throughput to workload. Gomez *et al.* [82] evaluated the performance of BLE when applying compression to sensing data before transmission to an IoT server. The electrocardiogram (ECG) data in health care monitoring is transmitted to the IoT server via BLE with enhanced techniques such as signal compression to avoid or reduce the transmission overload problem and in turn help energy conservation for devices. If raw data were transferred to the IoT server, it would cause delay and energy consumption, and thereby making a health monitoring system experience unacceptable quality.

Although the previous solutions considered interesting issues such as a neighbor discovery procedure in BLE and data compression, they did not try to optimize BLE duty cycling features (e.g., BLE Sniff subrating) during normal operation.

**Table 3:** Energy conserving solutions for WPAN-based IoT.

Category	Solution	Approach	Scheme	Metric	Control	Evaluation
IPv6 overhead/Master slave connectivity interval determination	[92]	Provided an energy consumption evaluation of BLE	Transmit power	Energy efficiency	Centralized/Distributed	Simulation
	[83]	Measured lifetime of BLE slave connected to master	Duty cycling	Energy efficiency, latency	Centralized/Distributed	Real time experiment
IPv6 Support in BLE	[79,80,81,92]	Solutions supporting IPv6 over Bluetooth LE	Transmit power	Energy efficiency, throughput, delay	Centralized/Distributed	Real time experiment
Health Care and Home Automation Implementation	[82]	BLE implementation for continuous data transmission	Data rate	Energy efficiency, delay	Centralized/Distributed	Real time experiment
	[84]	BLE implementation to save energy in home based control system	Transmit power	Energy efficiency, delay	Centralized/Distributed	Real time experiment
Z-Wave energy saving with fault tolerance	[89]	Automatic route reconstruction in case of failure	Fault tolerance	Energy efficiency	Distributed	Simulation
	[90]	Used a strip to store duplicated services	Fault tolerance	Energy efficiency	Distributed	Real time testbed
Z-Wave for efficient home automation / smart home implementation	[87,88]	Z-Wave deployment in home automation and smart homes	Remote control	Energy efficiency	Centralized/Distributed	Real time experiment

#### 4.3.2. Energy Conserving Solutions for Z-Wave

Z-Wave is one of the most widely used technologies in smart home IoT environments owing to its network reliability and stability. Authors in [82,87] considered different layers of Z-wave for more reliable and delay tolerant transmission, and efficient routing for efficient home management. Particularly, the MAC layer is enhanced with energy saving support, where devices use acknowledgment based collision avoidance techniques to reduce the chances for collision and avoid power waste.

Shih *et al.* [89] presented a solution to the problem of network faults caused by device failures. They proposed the use of a meta-routing framework which enables automatic route reconstruction at run-time to overcome faults by making devices keep track of their neighbors. If a node is facing any failure, it can be recovered by using the routing table of its nearby neighbors. For a low energy policy, the implementation of this framework can show energy efficiency for M2M communications.

We note that the discussed solutions did not consider the usage of sleeping intervals which could be a major source of energy conservation in devices. They did not consider possible improvements to Z-Wave routing which assumes a central controller with global topology information and thus may be a limitation in a network lifetime.

#### 4.3.3. Summary

Table 3 provides information about categorization of various energy conserving solutions for WPAN-based IoT, their approaches, and technical criteria such as schemes, metrics, control, and evaluation.

## 5. Discussion

In this section, we suggest a few research directions for energy conservation in wireless networking-based IoT. Though not conceptually new, these proposed directions can be interesting topics in the realization of IoT. Firstly, previous research on energy conservation in IoT mostly focused on single-radio data transmission. However, for a coverage extension purpose for IoT devices, IoT gateways may support the simultaneous operation of multiple heterogeneous radio interfaces to relay data to IoT servers. For example, in the latest IoT platform development by Open Interconnect Consortium [97] and AllSeen Alliance [98], gateways typically use a low power short range radio to communicate with nearby IoT devices and also use a long range radio to communicate with an IoT server. In a case that such gateways are battery-operated like Ericsson’s capillary network [99], energy conservation in utilizing multiple radio interfaces may need further investigation along with co-channel and adjacent channel interference issues.

For the coexistence of heterogeneous wireless networking standards for IoT, Software Defined Radio (SDR) can be a promising approach. Aust *et al.* [100] presented IEEE 802.11ah-based Software Defined Radio (SDR) prototype. In that prototype, authors provided a demo for evaluating the efficiency of upcoming 802.11ah WLAN protocol that supports long outdoor range (above 1 km) at moderate data rates (up to 100 Kbps). Lin *et al.* [101] proposed a wireless IoT platform based on SDR technologies. They demonstrated interference mitigation scenario at a base station by using a cognitive radio technology. Tragos *et al.* [102] discussed that an SDR framework can overcome the heterogeneity of IoT devices by seamlessly adapting their communication technology and creating wireless cells that provide access to heterogeneous devices. Energy efficiency for SDR-based IoT gateways and devices needs investigation.

Another direction regarding energy conserving IoT is based on energy generation. One of them is energy harvesting [103,104], in which IoT device energy can be fetched from outside environments like solar energy, wind energy, or some other types of energies. It can increase the lifetime of IoT devices up to years without any need of changing batteries. Another approach, in its early research phase, is battery recharging of IoT devices from radio signals [105].

The support of IoT devices in cellular wireless mobile networks has recently drawn significant interests from major mobile network operators [106]. In addition, IoT is considered as one of the essential components in development of the next generation 5G mobile network standards (3GPP Release 14 and later). The usage of cellular radio technologies can benefit from the exploitation of the well-established core network infrastructure without any concern about backhaul cost, but it comes at the sacrifice of power consumption for longer range transmission. Small cell solutions such as pico-cells and femto-cells that are expected to proliferate in future may result in significantly lower power consumption. Energy efficiency for the next-generation cellular radio-based IoT will also be an important research topic in future.

## 6. Conclusions

This paper has provided a comprehensive survey on energy conserving issues and solutions for battery-operated IoT devices from wireless networking aspects. The extant solutions have tackled various operational aspects of IoT devices, including the adjustment of duty cycles, collision/congestion avoidance schemes, mechanisms to manage device sleep time by switching off radios or increasing a standby time, efficient radio resource scheduling, the intelligent selection of heterogeneous radio interfaces, and so on. The real adoption of the solutions onto IoT devices should consider a combination of incorporated wireless radio access technologies. This survey has examined the literature regarding emerging IoT technologies and their energy conserving issues from a specific perspective of wireless networking. It will add to other recent surveys on IoT such as energy efficient multimedia streaming [107], communication standard bodies [108], and IoT semantics [109].
